# Evolutionary conservation analysis of human arachidonic acid metabolism pathway genes

**DOI:** 10.1093/lifemedi/lnad004

**Published:** 2023-02-10

**Authors:** Cengfan Wei, Meng Wang, Xiu-Jie Wang

**Affiliations:** Institute of Genetics and Developmental Biology, Innovation Academy of Seed Design, Chinese Academy of Sciences, Beijing 100101, China; University of Chinese Academy of Sciences, Beijing 100049, China; Institute of Genetics and Developmental Biology, Innovation Academy of Seed Design, Chinese Academy of Sciences, Beijing 100101, China; Institute of Genetics and Developmental Biology, Innovation Academy of Seed Design, Chinese Academy of Sciences, Beijing 100101, China; University of Chinese Academy of Sciences, Beijing 100049, China

**Dear Editor,**

Arachidonic acid (AA) is an essential fatty acid and one of the most abundant polyunsaturated fatty acids (PUFA) in all mammalian cells. It is typically esterified to membrane phospholipids and mainly released upon activation of cytosolic phospholipase A2 (cPLA2) to exert biological functions [1]. AA is mainly metabolized by three classes of key enzymes, namely cyclooxygenases (COX), lipoxygenases (LOX), and cytochrome P450 (CYP450), to form a spectrum of bioactive mediators [2]. The COX pathway is the first reported AA metabolizing pathway, which generates prostaglandins (PGs) and thromboxane A2 (TXA2) by the catalysis of COX-1 (PTGS1) and COX-2 (PTGS2) enzymes. The LOX pathway is catalyzed by several lipoxygenases and generates leukotrienes (LTs), lipoxins (LXs), as well as several hydroxyeicosatetraenoic acids (HETEs) (i.e., 5-HETE, 8-HETE, 12-HETE and 15-HETE). The CYP pathway has both ω-hydroxylase activity and epoxygenase activity [3], which can metabolize AA to generate hydroxyeicosatetraenoic acids (HETEs) (i.e., 19-HETE and 20-HETE) and epoxyeicosatrienoic acids (EETs) (i.e., 5,6-EET, 8,9-EET, 11,12-EET and 14,15-EET) [2].

With such diversified metabolic products, AA pathway has been proven to play key roles in many physiological processes (Wei et al., 2021), abnormal AA metabolism could lead to various diseases, such as cardiovascular diseases, metabolic diseases, inflammation and cancer. In general, both the COX and LOX pathway metabolites involve in the regulation of platelet aggregation, pain perception, immune modulation and other inflammatory related responses [4], whereas the CYP450 pathway is mainly restricted to liver and responsible for detoxification and drug metabolism [5, 6]. Despite extensive functional studies on the AA metabolism pathway genes, one intriguing question remains, that is how the emergence of these genes during evolution correlates with the acquirement of new functions in different species.

To address the above question, we systematically analyzed the sequence conservation of human AA metabolism pathway genes. We first collected AA pathway genes from the KEGG pathway database (Release 102.0, April 1, 2022). Among the 40 identified genes, 13 belonged to the COX pathway, 16 were involved in the LOX pathway, and 11 were assigned to the CYP pathway (Table S1; Fig. 1A). Gene Ontology (GO) analysis revealed that besides icosanoid metabolic related processes, the AA metabolism pathway genes were also enriched in xenobiotic stimulus response, cellular oxidation, inflammation and drug metabolism related biological processes (Fig. 1B). We next analyzed the sequence conservation status of these genes using the NCBI HomoloGene database (https://www.ncbi.nlm.nih.gov/homologene) and Protein BLAST database (Fig. 1C). Basically, protein sequences of key enzymes in the COX pathway are conserved from Euteleostomi. In the LOX pathway, the 5-LOX pathway is conserved throughout Eukaryota, the 12-LOX pathway is conserved from Opisthokonta, but the 15-LOX pathway emerged late and is only conserved from Amniota. For the CYP pathway, the ω-hydroxylase pathway is conserved from Euteleostomi, whereas the epoxygenase pathway is conserved from Boreoeutheria (Fig. 1C and 1D).

**Figure 1.**
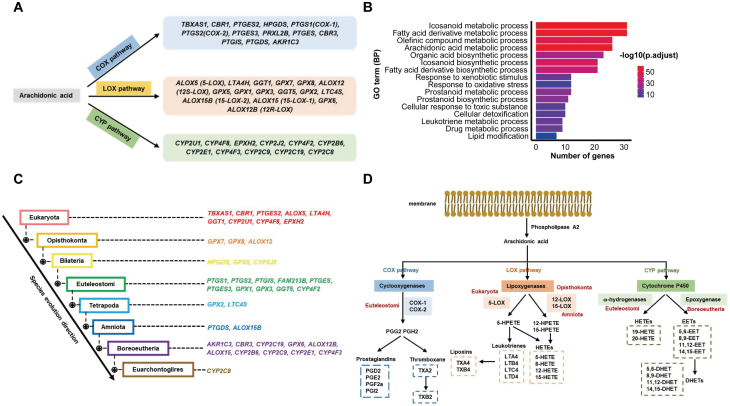
**Evolutionary conservation of human AA metabolism pathway genes.**(A) List of genes in the COX, LOX and CYP pathways. (B) Gene Ontology (GO) analysis of AA metabolism pathway genes. Shown are the enriched GO terms of the biological process category, the *X* axis represents the number of AA metabolism pathway genes belonging to each GO term. (C) The conservation status of AA metabolism pathway genes during evolution. Genes are shown at corresponding evolutionary tree branches where the sequence conservation was first observed. (D) Major metabolites of AA metabolic pathway and the conservation status of each gene in the pathway. The corresponding evolutionary branch in (C) is shown next to each gene. Specifically, Opisthokonta is for 12-LOX and Amniota is for 15-LOX.

The COX pathway is the first reported AA metabolism pathway, which converts AA into prostaglandins (PGs) and thromboxane A2 (TXA2). Functions of thromboxane A2 are opposite to those of prostaglandins, both play key roles in the regulation of platelet aggregation and vasoconstriction, and the dynamic balance of prostaglandins and thromboxane A2 are important for cardiovascular system homeostasis [7]. In addition, prostaglandins are also important pro-inflammatory mediators, especially during the pro-inflammatory stage. Among enzymes encoded by the 13 COX pathway genes (Fig. 2A), COX-1 (PTGS1) and COX-2 (PTGS2) are the most upstream key enzymes. The expression of *PTGS1* is constitutive, whereas the expression of *PTGS2* needs to be induced by hormones or inflammatory stimuli. The sequences of both PTGS1 and PTGS2, as well as their direct downstream enzymes (PTGIS, PRXL2B, PTGES, and PTGES3) are conserved from Euteleostomi to human (Fig. 2B). These enzymes mainly involve in the production of prostanoids, including PGE2, PGF2a, PGI2 and TXA2. Among these enzymes, PRXL2B participates in cellular oxidant detoxification, whereas other enzymes participate in bone development process, these functions are in concert with the development of skeletons in Euteleostomi. Interestingly, TBXAS1, CBR1, and PTGES2, which all involve in redox reaction, are conserved in Eukaryota (Fig. 2B), suggesting that a new antioxidant defense mechanism may emerge in eukaryotes compared to prokaryotes due to the appearance of mitochondria in eukaryotes. HPGDS, which generates PGD2 and its downstream metabolites, is conserved in Bilateria (Fig. 2B). Studies have shown that PGD2 participates in the regulation of motor behavior and sleep-wake cycle, thus may promote the development of nervous system, especially head. Downstream of HPGDS, PTGDS is conserved in Amniota (Fig. 2B). PTGDS may act as scavengers of harmful hydrophobic molecules, which is in concert with the adaptation to relatively dry terrestrial environment. AKR1C3 and CBR3 are both conserved in Boreoeutheria (Fig. 2B). AKR1C3 participates in keratinocyte differentiation, and CBR3 participates in cognition process, these functions may facilitate animals to be more adaptive to terrestrial life. 

**Figure 2.**
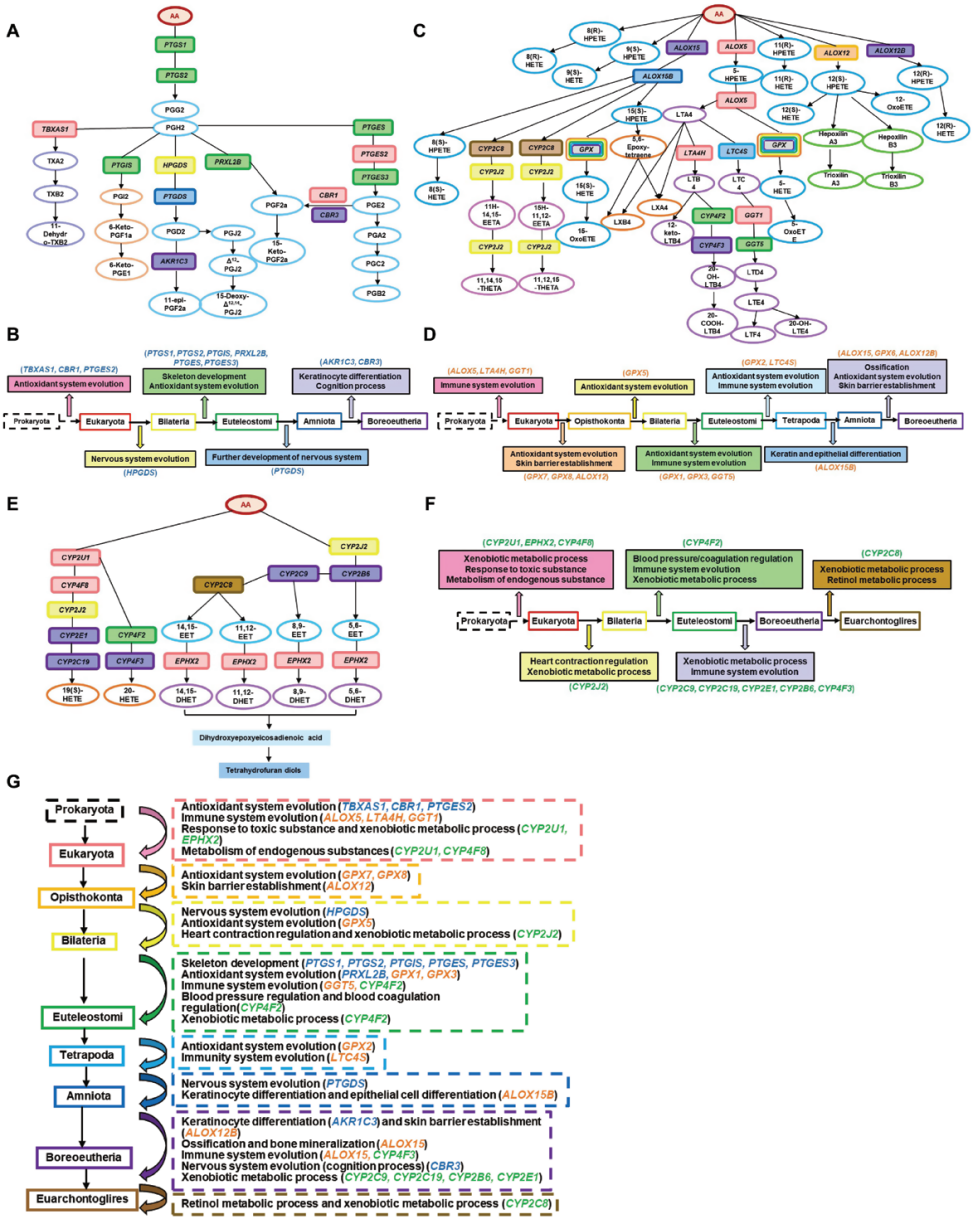
**Conservation status of each AA metabolic pathway genes.**(A) Conservation status of the COX pathway genes. Genes are presented as solid boxes and are shown in the same color as the evolutionary tree branch in which the sequence conservation starts. Metabolites are presented as ovals, and similar metabolites are shown with the same color. (B) Potential functions of the COX pathway genes in driving neofunctionalization during evolution. Boxes with solid colors present functions evolved with the emergence of evolutionary branches outlined by the same color. The evolutionary functional emergence information shown in (B) is inferred from literatures. (C) and (D), Conservation status and neofunctionalization analysis of the LOX pathway genes. (E) and (F), Conservation status and neofunctionalization analysis of the CYP pathway genes. Data presentation rules in (C)–(F) are the same as in (A) and (B). (G) Summary of inferred potential functions for AA metabolic pathway genes throughout evolution. Genes in the COX pathway, LOX pathway and CYP pathway are shown in blue, orange, and green colors, respectively.

The LOX pathway is responsible for metabolizing AA to leukotrienes (LTs), lipoxins (LXs) and hydroxyeicosatetraenoic acids (HETEs). Leukotrienes are a class of inflammatory mediators and important chemoattractants and agonists for leukocytes. Lipoxins are a class of endogenous lipid mediators with anti-inflammation, immune response regulation and cell damage repair effects, known as the “brake signal” of inflammatory response. HETEs (12-HETE and 15-HETE) mainly function in regulating vasocontraction and vasodilation, as well as the remodeling of blood vessels. Among the 16 LOX pathway enzymes (Fig. 2C), ALOX5, LTA4H and GGT1, which regulate the generation of LTA4, LTB4 and LTD4, are conserved in Eukaryota and may play roles in the establishment of immune system in eukaryotes (Fig. 2D). ALOX12 and ALOX12B, both involve in skin barrier development process, are conserved in Opisthokonta and Boreoeutheria, respectively (Fig. 2D). The conservation status of ALOX15 is the same as that of ALOX12B, whereas ALOX15B is conserved since Amniota (Fig. 2D). Majority enzymes in the LOX pathway belong to the GPX family, they are conserved in Opisthokonta (GPX7 and GPX8), Bilateria (GPX5), Euteleostomi (GPX1 and GPX3), Tetrapoda (GPX2), Boreoeutheria (GPX6), respectively (Fig. 2D). All these GPX family enzymes have antioxidant related functions, indicating the gradual development of antioxidant response system of animals during evolution.

The third AA metabolism pathway is the CYP pathway (Fig. 2E), which generates epoxyeicosatrienoic acids (EETs), dihydroxyeicosatrienoic acids (DHETs), 19(S)-hydroxyeicosatetraenoic acid (19(S)-HETE) and 20-HETE. EETs mainly function in vasodilation and inhibiting vascular inflammation, whereas 20-HETE functions in promoting vasoconstriction and vascular inflammation, both of which promote angiogenesis [8]. Enzymes involved in this pathway are mainly chromosome P450 (CYP) superfamily proteins, except for EPHX2 which converts EETs to DHETs. The most prominent function of the CYP pathway is to metabolize xenobiotics. Although the sequences of different CYP pathway members are conserved from different phylogenetic tree branches, they all participate in xenobiotic metabolic processes (Fig. 2F). In addition, CYP4F8 also participates in the metabolism and synthesis of endogenous substances (Fig. 2F), including PUFAs, cholesterol and prostaglandins. CYP2J2 is involved in the generation of HETEs and EETs, and participates in heart contraction regulation (Fig. 2F), which is in concert with the emergence of heart in Bilaterians. Interestingly, CYP4F2, which is involved in 20-HETE generation and conserved in Euteleostomi, participates in the regulation of blood pressure and blood coagulation (Fig. 2F), both functions are required after the emergence of heart. CYP4F3, conserved in Boreoeutheria, participates in the metabolic process of leukotriene B4 and inflammatory responses (Fig. 2F). Conserved in Euarchontoglires, CYP2C8 is involved in EET generation and participates in xenobiotic and retinol metabolism (Fig. 2F), functions of CYP2C8 include to maintain vision, to promote bone and tooth growth, and to increase neuroplasticity and neurogenesis. All these functions may contribute to the development of an advanced level of intelligence in Euarchontoglires. CYP450 family enzymes are also key players for drug metabolism, we found that for CYPs in the AA metabolism pathway, those conserved in Boreoeutheria are more studied in drug metabolism than the ones conserved from older species.

AA metabolism pathway plays important roles in many physiological processes as well as drug metabolism. Here, we systematically analyzed the sequence conservation of AA metabolism pathway genes, and identified clues linking AA metabolism pathway genes and the acquirement of new functions in different species throughout evolution (Fig. 2G). For example, the expansion of AA metabolism pathway and the conservation of the corresponding enzymes are well correlated with the emergence of antioxidant system in Eukaryota, the development of skeletons in Euteleostomi, and the more advanced nervous system in Boreoeutheria and Euarchontoglires. In addition, we also analyzed the functional conservation of these AA genes among human, mouse and rat by literate search, and found conserved functions for all 20 genes with available literatures. This information could help to better understand the driving force of gene sequence evolution and the functions of different members in the AA metabolism pathway, and may also shed light on AA metabolism related disease treatment and drug discovery.

## Research limitations

The potential roles of AA pathway genes in neofunctionalization during evolution revealed in this study were inferred from the species conservation information and literature searches, further experimental studies are desired to support these speculations.

## Supplementary Material

lnad004_suppl_Supplementary_Materials
