# Supplementary material for: Evolutionary conservation analysis of human arachidonic acid metabolism pathway genes
Source: Life Med. 2023 Feb 10;2(2):lnad004. doi: 10.1093/lifemedi/lnad004 (PMC11749852; doi:10.1093/lifemedi/lnad004)
Supplement: lnad004_suppl_Supplementary_Materials [file lnad004_suppl_Supplementary_Materials.pdf]

Supplementary Materials

Evolutionary conservation analysis of human arachidonic acid metabolism pathway genes

Methods

Data collection

The human arachidonic acid (AA) metabolism pathway was constructed according to the AA metabolism map in the KEGG pathway database (https://www.kegg.jp/pathway/map=hsa00590&keyword=arachidonic%20acid). Genes involved in the AA metabolism pathway were also collected from the KEGG database (https://www.kegg.jp/entry/hsa00590).

Gene conservation analysis

Gene conservation analysis was performed using information provided by the HomoloGene database (https://www.ncbi.nlm.nih.gov/homologene) andNCBI Prote BLAST database (https://blast.ncbi.nlm.nih.gov/Blast.cgi?PROGRAM=blastp&PAGE_TYPE=Bla stSearch&LINK_LOC=blasthome). The species conservation data for AA metabolic pathway genes were retrieved from the HomoloGene database using gene symbols as queries. The evolutionary conservation status of each gene was further verified using the Protein BLAST database, with E-value < $1e^{-6}$ and percent identity > 30% as the threshold for homology gene identification, and the maximum number of aligned

sequences to display was set as 5000. The Protein BLAST analysis results were in agreement with the sequence conservation information in the HomoloGene database for all analyzed genes.

Species evolution tree construction

The Taxonomy Common Tree tool (https://www.ncbi.nlm.nih.gov/Taxonomy/CommonTree/wwwcmt.cgi) in the NCBI Taxonomy database (https://www.ncbi.nlm.nih.gov/taxonomy) was used to construct species evolution tree with default settings, using the species conservation status of each gene as the input.

Gene Ontology analysis

Gene Ontology (GO) analysis was performed with clusterProfiler package in R and the online Metascape software (https://metascape.org/gp/index.html#/main/step1). Raw *p*-value was adjusted by the Benjamini-Hochberg method, using q-value < 0.05 as the threshold to select enriched GO terms. Only GO terms with more than 7 input genes were considered.

Functional analysis of AA metabolic pathway genes

The known functions of AA metabolism pathway genes were collected from the GeneCards database (https://www.genecards.org) and thorough literature searches, the neofunctionalization roles of each gene were inferred by combining its function and evolutionary conservation information. List of related literatures is shown in Table S2.

Data availability

Data analysis methods for this work are available in the online Supplementary Materials file. The list of AA metabolism pathway genes is shown in Table S1, and references used for neofuntionalization analysis of each AA metabolism pathway gene are shown in Table S2.

Table S1. List of arachidonic acid metabolism pathway genes.

Pathway	Gene symbol	Description
COX	PTGS1	Prostaglandin-endoperoxide synthase 1
	PTGS2	Prostaglandin-endoperoxide synthase 2
	PTGES	Prostaglandin E synthase
	PTGES2	Prostaglandin E synthase 2
	PTGES3	Prostaglandin E synthase 3
	CBR1	Carbonyl reductase 1
	CBR3	Carbonyl reductase 3
	PRXL2B	Peroxiredoxin like 2B
	TBXAS1	Thromboxane A synthase 1
	PTGDS	Prostaglandin D2 synthase
	HPGDS	Hematopoietic prostaglandin D synthase
	AKR1C3	Aldo-keto reductase family 1 member C3
	PTGIS	Prostaglandin I2 synthase
LOX	ALOX5	Arachidonate 5-lipoxygenase
	ALOX12	Arachidonate 12-lipoxygenase, 12S type
	ALOX12B	Arachidonate 12-lipoxygenase, 12R type
	ALOX15	Arachidonate 15-lipoxygenase
	ALOX15B	Arachidonate 15-lipoxygenase, type B
	LTA4H	Leukotriene A4 hydrolase
	LTC4S	Leukotriene C4 synthase
	GGT1	Gamma-glutamyltransferase 1
	GGT5	Gamma-glutamyltransferase 5
	GPX1	Glutathione peroxidase 1
	GPX2	Glutathione peroxidase 2
	GPX3	Glutathione peroxidase 3
	GPX5	Glutathione peroxidase 5
	GPX6	Glutathione peroxidase 6
	GPX7	Glutathione peroxidase 7
	GPX8	Glutathione peroxidase 8
CYP	CYP2U1	Cytochrome P450 family 2 subfamily U member 1
	CYP2J2	Cytochrome P450 family 2 subfamily J member 2
	CYP4F2	Cytochrome P450 family 4 subfamily F member 2
	CYP4F3	Cytochrome P450 family 4 subfamily F member 3
	CYP4F8	Cytochrome P450 family 4 subfamily F member 8
	CYP2E1	Cytochrome P450 family 2 subfamily E member 1
	CYP2C8	Cytochrome P450 family 2 subfamily C member 8
	CYP2C9	Cytochrome P450 family 2 subfamily C member 9
	CYP2C19	Cytochrome P450 family 2 subfamily C member 19
	CYP2B6	Cytochrome P450 family 2 subfamily B member 6
	EPHX2	Epoxide hydrolase 2

Table S2. References used for neofuntionalization analysis of arachidonic acid metabolism pathway genes.

Pathway	Gene	PMID
	PTGES2	18824665
	TBXAS1	29340222
	CBR1	19442656, 30376862,
	HPGDS	10781097
	PRXL2B	18006499, 21873635
**COX**	PTGS1	30760327, 33800915
	PTGS2	29515175, 17352658,
	PTGES/PTGES3	33129574, 18163499
	PTGIS	34260805, 24780398
	PTGDS	20667974, 9475419
	AKR1C3	22170488
	CBR3	23227193
	LTA4H	11675384, 33329520,
	GGT1	14754911, 21447318
	ALOX5	17114001, 23246375,
	GPX	23201771
**LOX**	ALOX12	23578768
	GGT5	30842656, 21873635
	LTC4S	9153254, 12432940
	ALOX15B	11839751, 12432921
	ALOX15	14716014, 16799083
	ALOX12B	24070899
	EPHX2	33925035, 12869654
	CYP2U1	15987645
	CYP4F8	10405341
	CYP2J2	8631948, 12869654, 16839864
**CYP**	CYP4F2	18391101, 19297519,
	CYP2B6/CYP2C9/CYP2C19/CYP2E1	19029318, 34884615, 28480783, 10422890
	CYP2C8	10484078, 18413310, 3260878, 27830507,
